# Psychoactive substance abuse among commercial bus drivers in Umuahia, Abia State, South-Eastern Nigeria: an uncontrolled “epidemic” with attendant road traffic crashes

**DOI:** 10.1186/s12889-023-15039-6

**Published:** 2023-02-06

**Authors:** Roseline Oluyemisi Akande, Joel Olufunminiyi Akande, Olaniyan Akintunde Babatunde, Adeola Olajumoke Ajayi, Akindele Amos Ajayi, Roseline Olabisi Ige, Ajedotun Shittu Saliu, Abayomi Akande, Muideen Babatunde Olatunji

**Affiliations:** 1 Department of Community Medicine, LAUTECH, Ogbomoso, Oyo State Nigeria; 2 Department of Chemical Pathology, LAUTECH, Ogbomoso, Oyo State Nigeria; 3 Oyo State Primary Health Care Board, State Secretariat, Agodi, Ibadan, Oyo State Nigeria; 4 Oriire Local Government Health Authority, Ikoyi-Ile, Oyo State Nigeria; 5 Department of Psychiatry, Uniosun Teaching Hospital, Osogbo, Osun State Nigeria; 6 Department of Family Medicine, Uniosun Teaching Hospital, Osogbo, Osun State Nigeria; 7 Department of Community Medicine, LAUTECH Teaching Hospital, Ogbomoso, Oyo State Nigeria; 8 Al Kamil General Hospital, Al Kamil, Makkah Province Saudi Arabia; 9 Department of Community Medicine, Faculty of Public Health, College of Medicine, University of Ibadan, Oyo State Ibadan, Nigeria

**Keywords:** Substance abuse, Bus Drivers, Road traffic crashes, Commercial

## Abstract

**Background information:**

The unprecedented depletion of the productive workforce has been majorly attributed to road traffic crashes (RTCs). The attendant consequences of this depletion have been found to constitute a serious global public health challenge, with the use of psychoactive substances among drivers implicated in every three of five motor vehicle accidents. Hence, this study assessed the pattern and explored the determinants of substance abuse among commercial bus drivers in Umuahia, Abia State.

**Methods:**

A descriptive cross-sectional design was used for the study. Four hundred commercial bus drivers were recruited from selected motor parks in Abia-state, using a multistage sampling technique from October to December 2020. A pretested, interviewer-administered questionnaire was administered to obtain socio-demographics and information on substance abuse. Data were analyzed using IBM SPSS version 25; bivariate analysis was done using Chi-square. The level of significance was at 5%.

**Results:**

The mean age of the respondents was 40.03 ± 10.50 years. The proportion of respondents who had ever abused a psychoactive substance was 74.6%. The most commonly abused substances among drivers include alcohol (51%), cigarettes (27%), and alcoholic herbal mixtures (16%). The study participants had poor knowledge (54.5%) and poor perception (63.2%) about psychoactive substance abuse. Among the factors found to be significantly associated with substance abuse among respondents were ethnicity (*p* = 0.002), religion (*p* = 0.009) and monthly income (*p* = 0.013) of the respondents, poor knowledge (*p* < 0.001) and poor perception (*p* < 0.001). However, this study found religion (*p* = 0.031; OR = 5.469; CI = 1.170 to 25.555), knowledge (*p* < 0.001; OR = 4.21; CI = 2.201 to 8.287) and perception (*p* < 0.001; OR = 9.828; CI = 15.572 to 65.052) as factors that were associated with the higher likelihood of psychoactive substance abuse.

**Conclusion:**

Religion, poor knowledge and perception were associated with the higher likelihood of psychoactive substance abuse among commercial bus drivers. Targeting commercial bus drivers for educational interventions and using religious leaders as conveyor belts may reduce the use of psychoactive substances among them.

## Background information

According to the World Health Organization, substance abuse refers to the harmful or hazardous use of psychoactive substances, including alcohol and illicit drugs. In contrast, substance use refers to the use of selected substances, including alcohol, tobacco products, drugs, inhalants, and other substances that can be consumed, inhaled, injected, or otherwise absorbed into the body with possible dependence and other detrimental effects [1]. Abuse can result from using a substance in a way that is not intended or recommended or from using more than prescribed. In other words, someone can use substances and not be addicted or even have a substance use disorder [2, 3]. The critical impacts of illicit drug use on society vary from physical to psychological and social harm to the individual and others affected by the behaviour of the substance abuser [3].

In 2021, the United Nations Office on Drugs and Crime (UNODC) estimated that about 5.5 per cent of the population aged between 15 and 64 years had used drugs at least once in the past year, while 36.3 million people who use drugs suffer from drug use disorders [3]. Road traffic accidents (RTAs) have been on an alarming increase and constitute a serious public health problem across the globe, causing over 1.2 million deaths annually and about 20–50 million injuries [4]. In many developing countries, driving under the influence of drugs (DUI) also constitutes a major public health burden, putting both the drivers and the passengers, as well as other road users, at risk of injuries and deaths, with more than 85% of all deaths and 90% of disability-adjusted life years lost from road traffic injuries [5, 6].

In 2018, the prevalence of any drug use in Nigeria was estimated at 14.4 per cent of the population aged between 15 and 64. Also, the extent of drug use in Nigeria is comparatively high compared with the 2016 global prevalence of any drug use of 5.6 per cent among the adult population [7]. Substance abuse in Nigeria spreads across different age groups, sex, socio-economic classes and professions, especially drivers [8]. Previous studies in Nigeria established that most commercial drivers use drugs to stay awake at night (kolanut and coffee), to stay alert throughout the day (cigarettes, cannabis), as a way to relax (alcohol), and to reduce pain (aspirin) during their busy and stressful work schedules [6, 8]. In Nigeria, Federal Road Safety Corps (FRSC) statistics indicated 5,157 lives were lost in road traffic accidents in three years out of 18,303 reported crashes [6]. Although the causes of motor vehicle accidents include a variety of factors such as bad driving, long work schedules, and unnecessary haste, psychoactive substances are said to play a significant role in three out of every five motor vehicle accidents [5, 6]. Intoxicants such as alcohol and marijuana have also been documented to affect the mental state of drivers, leading to altered perceptions, delayed reactions and increased risk for the occurrence of road traffic crashes (RTCs) [5]. It is common to observe public display and sale of non-commercial alcohol within and in the immediate environs of motor parks in Nigeria, giving drivers uncontrolled access to alcohol and other psychoactive substances in the line of duty. Oftentimes, they are sold to unwary buyers as herbal concoctions to cure ailments such as malaria or low back aches, popularly referred to as “jedi-jedi” in local parlance [9, 10].

In recent years, more attention has been drawn to psychoactive substance use and driving due to numerous road traffic accidents causally related to substance abuse [5]. Despite the intense campaigns against drunk-driving carried out by the Federal Road Safety Corp and other civil organizations, the use of psychoactive substances among commercial drivers has continued without check in Nigeria [11, 12]. Commercial bus drivers constitute an important occupational group, hence the need to focus on the influence of different factors that drive the rising prevalence of substance abuse among them. Additionally, there have been several agitations of secessions among different ethnic groups across Nigeria, especially among the people of Southeastern Nigeria, who felt cheated and marginalized in the scheme of things. Consequent to this struggle for self-determination, many clashes and violence have resulted in morbidity and mortality, which are more prevalent among commercial bus drivers. On record, the commercial bus drivers’ involvement is aided by the use and abuse of psychoactive substances. These clashes were found to impact the passengers and the communities negatively [13]. It is hoped that this study’s findings will help reawaken societal consciousness and inspire policymakers and law enforcement agents to devise strategies to combat the threat of psychoactive substance abuse among commercial bus drivers. Therefore, this study explored the pattern and factors associated with the use of psychoactive substances among commercial bus drivers in Umuahia with a view to reducing cognitive impairment resulting from abuse.

## Methods

### Study location

This study was carried out in Umuahia, the capital of Abia State, in South-eastern Nigeria. There are fifty-nine (59) commercial motor parks in Abia State, while twelve (12) are located in Umuahia. According to the National Union of Road Transport Workers (NURTW) Abia State chapter, there are 1,058 commercial drivers in Umuahia, Abia state.

### Study design and population

This was a descriptive cross-sectional study carried out between October and December 2020, among registered commercial drivers in motor parks in Umuahia, Abia State. The study population included all commercial bus drivers involved in inter-state travel.

Inclusion/exclusion criteria: Only interstate commercial bus drivers who have spent at least 6 months on the job were recruited into the study. Inter-state commercial bus drivers not registered with any transport union were excluded.

### Sample size determination

The minimum sample size was determined by the Cochran’s formula (Z^2^pq/e^2^) [14, 15]. The minimum sample size calculated was 362. After taking care of an anticipated non-response rate of 10%, a sample size of 400 respondents was obtained. Participants were recruited using a multi-stage sampling technique.

### Sampling technique

First stage: Umuahia comprises of two local government areas (LGAs) from which one LGA was randomly selected using a ballot technique. Second stage: A list of all the political wards was made in the selected LGA from which eight (8) wards were randomly selected using a ballot technique.

Third stage: A list of all the motor parks was drawn in each of the selected wards and one motor park was chosen by simple random sampling using a ballot technique making a total of 8 motor parks. Fourth stage: The proportional number of respondents interviewed in each of the selected motor parks was arrived at using proportionate allocation. A systematic random sampling technique was used to select eligible respondents in each of the selected motor parks. The first respondent was selected by simple random sampling through balloting, and all the eligible respondents in the motor parks were interviewed. Subsequently, every 3rd respondent was selected until the desired sample size was met. If respondents in a selected motor park declined, the respondents in the motor park with the next number replaced such.

### Research instrument and data collection methods

A semi-structured questionnaire was developed after thoroughly reviewing relevant literature to collect data. The questionnaire was used to collect relevant information on the socio-demographic characteristics of the respondents, their knowledge of substance abuse, and their perception of substance abuse among drivers.

### Validation and pre-test of research instrument

The validity and reliability of the questionnaire were determined before the final collection of data. Three Nigerian experts in the field of Epidemiology at a Nigerian university evaluated the extent to which the variables in the questionnaires were relevant to the study’s objectives. Thereafter, 40 questionnaires were pretested among eligible respondents in Aba South LGA, Abia-State, a LGA different from the study location. The responses provided helped to clarify instructions as well as modify questions or response categories where necessary. Cronbach’s alpha internal consistency reliability of 0.86 was achieved for the analyzed variables.

### Measurement of main outcome variables

The dependent variable was substance abuse, while the independent (predictor) variables were the socio-demographic factors (age, marital status, religion, educational status, and monthly income). The respondents’ knowledge was assessed using ten questions adapted from previous studies [8, 11, 16]. Every correct response got a score of 1, and an incorrect response was scored zero. The perception of individuals about psychoactive substance abuse was obtained using seven items on a five-point Likert scale ranging from strongly disagree (1) to strongly agree (5) [17]. The responses were scored 5,4,3,2, and 1 in that order for a good perception. The cut-off point (≥ 60%) was set as informed by similar studies [18, 19]. Composite scores were calculated for the individuals to determine the level of perception, and those scoring ≥ 60% were considered as having good perception.

### Data analysis

The questionnaires were sorted, entered into a computer, and the resulting data were analyzed with the Statistical Package for Social Sciences (SPSS) version 25. A descriptive analysis of all the variables measured was first done, and the categorical variables were reported as frequencies and percentages. At the bivariate level, cross-tabulations were done to test for associations, and the categorical variables were assessed using the Pearson Chi-square test. The multivariate analysis determined the determinants of substance abuse among commercial bus drivers using the logistic regression model. Only variables whose *p*-values were statistically significant, were entered into the model. The estimated coefficients were expressed as odds ratios (ORs) and their 95% confidence intervals were also calculated. The level of significance for the study was set at *p* < 0.05.

### Ethical Considerations

Ethical approval was obtained from the Abia State Ministry of Health with reference number MOH/AS/EC/19/20/1206. Permission to carry out the project was obtained from the Umuahia North Local Government Authorities as well as from the Chairmen and Secretaries overseeing the selected motor parks. Informed consent was taken from each participant after adequate explanation of the objectives of the study and the benefits of the study. Respondents were told that participation of persons is voluntary and all information gathered was kept confidential. The participants were identified using only serial numbers and data security were assured.

### Operational definition of variables

- A psychoactive substance user: defined as a person who used at least one of the psychoactive substances to keep awake while driving [4].
- Prevalence: defined as the frequency of study subjects who used psychoactive substances in the past month [20].
- Current use: consuming any substance within the last one month/30 days [21].
- The responses of the commercial bus drivers in this study were self-reported and have to be accepted as they were stated, the research assistants were well trained to minimize reporting bias. In addition to this, social desirability bias was envisaged and this was reduced by avoiding leading questions and using interval questions. We might not be able to generalize our findings to the whole country. However, the findings of our study could be extrapolated to the entire Southern part of Nigeria because of the similarities in socio-economic and cultural characteristics.: refers to the use of any of the substances at least once in an individual’s lifetime [21].
- A rest break between driving: defined as a 30 min break taken by a commercial driver after driving for a period of 8 cumulative hours without at least a 30-minute interruption [22].
- Psychoactive substance abuse: refers to the harmful or hazardous use of psychoactive substances, including alcohol and illicit drugs such as cannabis, amphetamines, cocaine, opioids, and non-prescribed psychoactive prescription medication [3].
- OR is defined as a “state” of periodic or chronic intoxication, detrimental to the individual and society, produced by the repeated consumption of a substance (natural or synthetic) [23].
- Alcohol abuse: For men, consumption of more than four drinks per day or more than 14 drinks per week. For women, consuming more than three drinks per day or more than seven drinks per week [24].
- Cigarettes abuse: Ever smokers were classified as those who had smoked at least 100 cigarettes in their lifetime or at least one cigarette/day for one year [25].
- Kolanuts abuse: Consumption of > 5 kola nuts per day [26].
- Coffee abuse: Consumption of > 5 cups of coffee per day within the past 30 days [27].
- Marijuana abuse: Daily use of marijuana by drivers in the past 30 days [28].

## Results

The association between the psychoactive substance abuse and the socio-demographic characteristics of the respondents is shown in Table 1. The proportion (73.1%) of the respondents who abused psychoactive substances was significantly higher among respondents of Igbo ethnic extraction (*p* = 0.002). In the religion category, the proportion (89.7%) was also significantly higher among commercial bus drivers who were Christians (*p* = 0.009). Respondents with monthly income of between N30, 000 and N39, 000 constituted a significantly higher proportion (80.7%) of the commercial bus drivers who abused psychoactive substance (*p* = 0.013).

**Table 1:** Association between socio-demographic characteristics and psychoactive substance abuse among respondents (*N* = 400)

Variables	Psychoactive substance abuse (%)		Statistics
	**Yes (*n* = 297)**	**No (*n* = 103)**	
**Age groups (in years)**			
< 30	52 (17.5)	13 (12.6)	χ^2^ = 3.520
30 – 39	98 (33.0)	37 (35.9)	df = 3
40 – 49	89 (30.0)	26 (25.2)	*p* = 0.318
≥ 50	58 (19.5)	27 (26.2)	
**Sex**			χ^2^ = 0.014
Male	289 (97.3)	100 (97.1)	df = 1
Female	8 (2.7)	3 (2.9)	*p* = 0.907
**Ethnicity**			
Yoruba	46 (15.1)	10 (9.7)	LR = 14.685
Igbo	217 (73.1)	91 (88.3)	
Hausa	32 (10.8)	2 (1.9)	df = 3
Others	2 (0.7)	0 (0.0)	*p* = 0.002*
**Religion**			
**Educational status**			
None	67 (22.6)	21 (20.4)	χ^2^ = 4.655
Primary	91 (30.6)	35 (34.0)	df = 3
Secondary	97 (32.7)	40 (38.8)	*p* = 0.199
Higher institution	42 (14.1)	7 (6.8)	
**Marital status**			
Single	87 (29.3)	26 (25.2)	LR = 5.626
Married	170 (57.2)	69 (67.0)	df = 4
Widowed	19 (6.4)	2 (1.9)	*p* = 0.229
Separated	13 (4.4)	3 (2.9)	
Divorced	8 (2.7)	3 (2.9)	
**Religion**			
Christianity	234 (78.8)	95 (92.2)	χ^2^ = 9.491
Islam	37 (12.5)	5 (4.9)	df = 2
Traditional worshipper	26 (8.8)	3 (2.9)	*p* = 0.009*
**Family setting**			χ^2^ = 2.355
Monogamy	233 (78.5)	88 (85.4)	df = 1
Polygamy	64 (21.5)	15 (14.6)	*p* = 0.125
**Monthly income (in naira)**			
10,000 – 19,000	33 (11.1)	13 (12.6)	χ^2^ = 12.667
20,000 – 29,000	92 (31.0)	22 (21.4)	df = 4
30,000 – 39,000	107 (36.0)	29 (28.2)	*p* = 0.013*
40,000 – 49,000	49 (16.5)	26 (25.2)	
≥ 50,000	16 (5.4)	13 (12.6)	

^*^ Statistically significant,  *LR*  Likelihood Ratio

Table 2 shows the pattern of substances of abuse among commercial bus drivers. This study revealed that about 99.5% (398) of the respondents had ever used a psychoactive substance, of which 74.6% (297) had ever abused it. Out of the respondents that had ever used any psychoactive substance, 50.3% (201) consumed alcohol, 30.5% (122) smoked cigarettes, and 21.3% (85) used alcoholic herbal mixtures. The study found that the substances currently used by the respondents’ ranged from alcohol 51% (204) to cigarettes 27.3% (109), alcoholic herbal mixtures 16% (64) and 7.8% (31) tramadol. About 37% (109) of the respondents made a personal decision to use psychoactive substances. 49.2% of the drivers (146) used the substances twice daily and 31.6% (94, 31.6%) used the substances before the commencement of driving activity. Of the respondents, 60.3% (179) could perform their duties optimally without using a psychoactive substance.

**Table 2:** Pattern of psychoactive substances of abuse among respondents (*N* = 400)

Variables	Frequency (*n*)	Percent (%)
**Ever used a psychoactive substance**		
** Yes**	398	99.5
** No**	2	0.5
**Ever abused a psychoactive substance (***n* = 398)		
Yes	297	74.6
No	101	25.4
^a^**Substances of abuse ever used by respondents**		
Alcohol	201	50.3
Cigarettes	122	30.5
Alcoholic herbal mixtures	85	21.3
Kolanut	47	11.8
Marijuana	42	10.5
Coffee	7	1.8
Tramadol	43	10.8
^a^**Substances currently used by respondents**		
Alcohol	204	51.0
Cigarettes	109	27.3
Alcoholic herbal mixtures	64	16.0
Kolanut	42	10.5
Marijuana	34	8.5
Coffee	7	1.8
Tramadol	31	7.8
**Person who introduced respondent to the substance (*n* = 297)**		
Personal decision	109	36.7
Colleagues	98	33.0
Friends	90	30.3
**Regularity of abuse of these substances (*n* = 297)**		
Once daily	60	20.2
Twice daily	146	49.2
Thrice daily	64	21.5
More than thrice	27	9.1
**Time of abuse of these substances (*n* = 297)**		
Before driving	94	31.6
During driving	75	25.3
After driving	58	19.5
Any time	70	23.6
**Optimal performance without substance (*n* = 297)**		
Yes	179	60.3
No	118	39.7

^a^Multiple responses allowed

Table 3 shows that 54.5% (218) of the respondents strongly agreed that the use of substances helped them to relieve stress, while 38.3% (153) strongly agreed that the substances helped them to relax and sleep after a hard day’s work. Approximately 39% of the drivers strongly agreed that using substances gives them pleasure and helps them work harder for more money.

**Table 3:** Respondents’ perception about psychoactive substance abuse (*N* = 400)

Variables	Perception (%)				
	**SA**	**A**	**I**	**D**	**SD**
Substances help to relief stress at work	218 (54.5)	69 (17.3)	75 (18.8)	31 (7.8)	7 (1.8)
Substances help to relax/sleep after a hard day’s job	153 (38.3)	108 (27.0)	89 (22.3)	41 (10.3)	9 (2.3)
Substances gives pleasure	155 (38.8)	71 (17.8)	125 (31.3)	39 (9.8)	10 (2.5)
Substances help to work hard to make money	133 (33.3)	80 (20.0)	111 (27.8)	60 (15.0)	16 (4.0)
Substances are due to peer group pressure	126 (31.5)	68 (17.0)	138 (34.5)	53 (13.3)	15 (3.8)
Substances help to suppress anxiety	128 (32.0)	72 (18.0)	122 (30.5)	60 (15.0)	18 (4.5)
Substances help to keep awake while driving	105 (26.3)	66 (16.5)	145 (36.3)	56 (14.0)	28 (7.0)

*SA* Strongly agree, A Agree, *I* Indifferent, *D* Disagree, *SD* strongly disagree

Table 4 shows the respondents’ knowledge about psychoactive substance abuse. About 395, (98.8%) of them correctly identified alcohol as a psychoactive substance, followed by cigarettes, 317 (79.3%) and herbal alcoholic mixtures 269 (67.3%) amongst others. When asked about the harmful consequences of psychoactive substance abuse, only few of the study participants 2, (0.5%) gave correct responses on increased risk-taking during driving, 264 (66.0%) gave correct responses on increased risk of road traffic accidents, while 290 (72.5%) gave correct responses on over estimation of driving skills of the driver.

**Table 4:** Respondents’ knowledge about psychoactive substance abuse (*N* = 400)

Variables	Correct responses *n* (%)	Incorrect responses *n* (%)
**The following are the commonly available substances of abuse**^a^		
Alcohol	395 (98.8)	5 (1.3)
Cigarettes	317 (79.3)	83 (20.8)
Herbal-Alcoholic mixtures	269 (67.3)	131 (32.8)
Cola nuts	217 (54.3)	183 (45.8)
Marijuana	199 (49.8)	201 (50.2)
Coffee	103 (25.8)	297 (74.3)
Tramadol	192 (48.0)	208 (52.0)
Prescription drugs	75 (18.8)	325 (81.3)
**Harmful consequences of substance abuse include: **^a^		
It increases risk taking during driving	2 (0.5)	398 (99.5)
It increases the risk of road traffic accidents	264 (66.0)	136 (34.0)
It leads to over- estimation of driving skills of the driver	290 (72.5)	110 (27.5)
It leads to ignoring road signs	182 (45.5)	218 (54.5)

^a^Multiple responses allowed

**Fig. 1:**
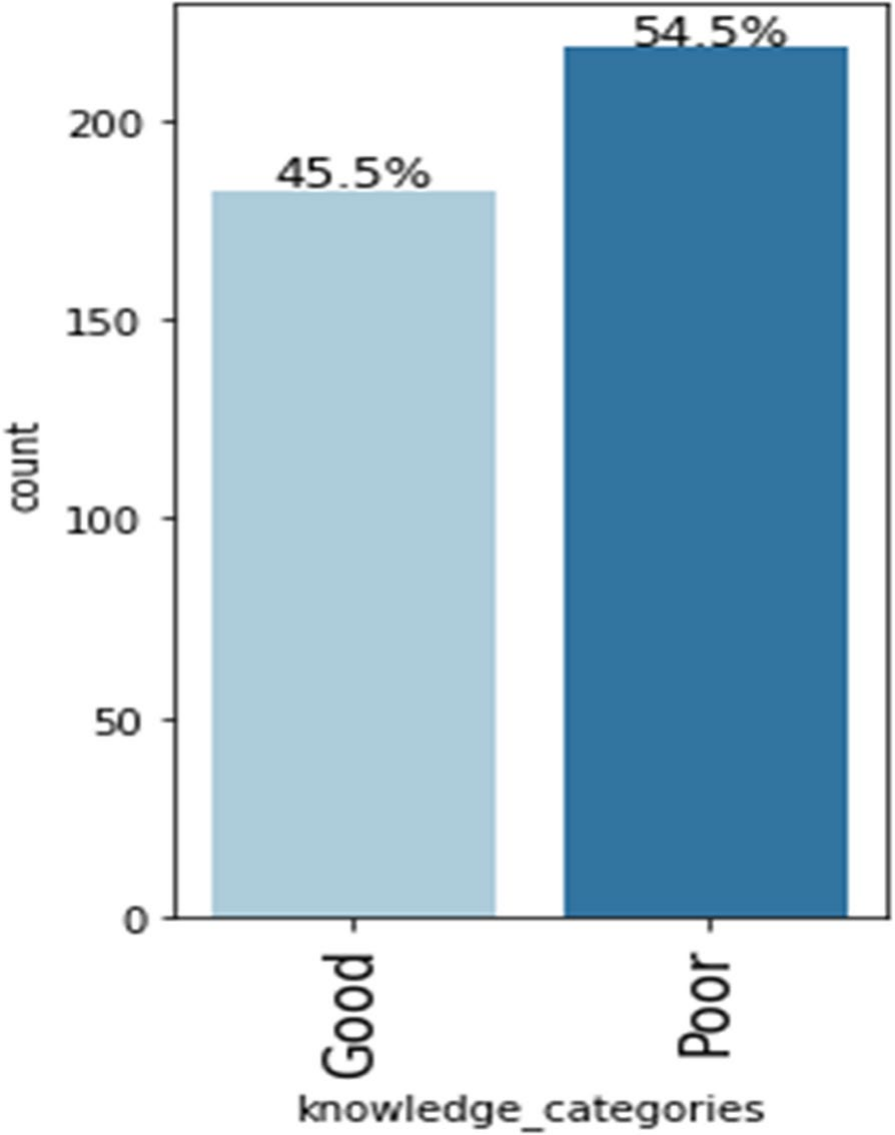
Overall knowledge about psychoactive substance of the commercial bus drivers in Umuahia, Abia State

Figure 1 shows the overall knowledge of the commercial bus drivers, dichotomised into good and poor knowledge. Over half of the study participants had poor knowledge (54.5%) while less than half of them (45.5%) had good knowledge about psychoactive substance abuse.

Table 5 shows the relationship between occupation profile, knowledge, perception, and psychoactive substance abuse. The proportion (61.6%) of the respondents who abused psychoactive substances was significantly higher among the respondents who had poor knowledge (*p* < 0.001). Respondents who had negative perception represented a significantly higher proportion (79.8%) of the drivers who abused psychoactive substances.

**Table 5:** Association between occupation profile, knowledge, perception and psychoactive substance abuse among the Respondents (*N* = 400)

Variables	Psychoactive substance abuse		Statistics
	**Yes (*n* = 297)**	**No (*n* = 103)**	
**Occupation status**			χ^2^ = 0.525
Full time	202 (73.2)	74 (26.8)	df = 1
Part time	95 (76.6)	29 (23.4)	*p* = 0.469
**Length of time working as a driver (in years)**			
0 – 4	28 (65.1)	15 (34.9)	χ^2^ = 7.204
5 – 9	71 (77.2)	21 (22.8)	df = 4
10 – 14	98 (79.0)	26 (21.0)	*p* = 0.125
15 – 19	72 (75.0)	24 (25.0)	
≥ 20	28 (62.2)	17 (37.8)	
**Knowledge about Substances of Abuse**			χ^2^ = 23.554
Poor	183 (83.9)	35 (16.2)	df = 1
Good	114 (62.6)	68 (37.4)	*p* < 0.001*
**Perception towards Substance Abuse**			
Poor	237 (93.7)	16 (6.3)	χ^2^ = 135.88
Good	60 (40.8)	87 (59.2)	df = 1
			*p* < 0.001*

There was no statistically significant relationship between the study participants’ occupation status (*p* = 0.469), length of time driving in years (*p* = 0.125), and substance abuse.

Table 6 shows the logistic regression of factors associated with psychoactive substance abuse among the respondents. Drivers who were Muslims had significantly lesser odds (18.3%) of psychoactive substance abuse compared with Christians (*p* = 0.031; OR = 0.183; CI = 0.039–0.855;). Respondents who had poor knowledge about psychoactive substance abuse were found to be 4 times more likely to indulge in psychoactive substance abuse (*p* < 0.001; OR = 4.27; CI = 2.201 to 8.287). Also, the study participants with poor perception towards substance abuse were 10 times more likely to be involved in psychoactive substance abuse (*p* < 0.001; OR = 9.828; CI = 15.572 to 65.052).

**Table 6:** Logistic regression of factors associated with psychoactive substance abuse among the respondents (*N* = 400)

Variables	*p*-value	Odds Ratio	95% Confidence Interval	
			**Lower**	**Upper**
**Ethnicity**				
Yoruba		1		
Igbo	0.883	1.080	0.388	3.008
Hausa	0.106	0.201	0.029	1.406
Others	1.000	0.000	0.000	0.000
**Religion**				
Christianity	0.031*	1	1.170	25.555
Islam	0.135	5.469	0.622	34.075
Traditional worshipper		4.602		
**Monthly income (in naira)**				
10,000 – 19,000	0.489	0.682	0.230	2.018
20,000 – 29,000	0.180	0.487	0.170	1.395
30,000 – 39,000	0.647	1.303	0.420	4.038
40,000 – 49,000	0.171	2.584	0.663	10.074
≥ 50,000		1		
**Knowledge about Substances of Abuse**				
Poor	< 0.001*	4.271	2.201	8.287
Good		1		
**Perception towards Substance Abuse**				
Poor	< 0.001*	9.828	15.572	65.052
Good		1		

^*^ Statistically significant

Area Under the Curve = 0.643

## Discussion

This study was aimed at assessing the determinants of psychoactive substance use among commercial bus drivers in Southeastern, Nigeria. The symmetry obtained in the age distribution of the respondents signified that we gathered information from the right and un-skewed group of people. This study’s overall lifetime prevalence of psychoactive substance use in this study was 74.3%. This finding is lower than previous findings in Kano, Nigeria (81.1%), Calabar, Nigeria (85.4%) and in Lokoja, Nigeria (93.8%) among commercial vehicle drivers [8, 29, 30]. The pattern of psychoactive substances abused by the respondents revealed that alcohol, cigarettes, alcoholic herbal mixtures, kolanuts, and marijuana were the most common agents. This further gives credence to the fact that abuse of psychoactive substances remains a regular occurrence among many commercial drivers which may occur during and out of the driving periods. This result is also consistent with a similar study in Lagos, Nigeria, whose study showed that alcohol, tobacco, and stimulants such as coffee and kolanut were the most prevalent substances used among their study participants [31]. The availability and accessibility of these psychoactive substances to the drivers in and around the motor parks should be checked by the National drugs and law enforcement Agency (NDLEA).

In terms of periodicity, most of the drivers in this study used these substances before and during driving engagements. This observation is quite worrying considering the increased risk of road traffic crashes from impaired judgments, fatigue, and stress while driving for several hours. Poor enforcement of laws prohibiting the sale of psychoactive substances within or around motor parks could encourage substance abuse among these drivers and should be of paramount importance in tackling this menace. Christopher Okpataku also observed a similar trend in his study conducted among commercial drivers in Kaduna, North West Nigeria [32]. The perception of the majority of the drivers in this study was that use of psychoactive substances helped them relieve stress after a long day at work, get more income, and gives them pleasure. Corroborating this result is the finding of Yunusa et al. in Kano, North-west Nigeria, whose study also showed that the desire to work hard for more income (48%), for pleasure (72%), and to relieve stress (81%) were the major reasons why the respondents engaged in substance abuse [8]. Hence, commercial bus drivers need to be constantly reminded using suitable channels that the abuse of psychoactive substances will not eliminate the stressors mentioned above. More so, abuse of these psychoactive substances repeatedly, could result in addiction and other chronic mental health conditions, ultimately affecting their job performance and ability to earn a livelihood.

In the current study, over half (54.5%) and more than two-thirds (63.2%) of the drivers had overall poor knowledge and poor perception about psychoactive substance abuse, respectively. A good knowledge and perception about the use of psychoactive substances and its side effects play important roles in the making of informed decisions that will eventually alter behaviour among abusers. A study conducted on the knowledge and attitude of drivers in Ghana on the use of alcohol showed that the majority of the drivers expressed an understanding that drunk driving was a significant risk for road traffic crashes. Despite this good knowledge, most of the drivers continued to use these drugs or substances blatantly ignoring the consequences [33]. Consequently, these identified deficits in knowledge, observed in this study, needs to be addressed by the relevant authorities, including non-governmental organizations (NGOs). Organizing workshops and training may help improve the drivers’ knowledge about the enormous risks that psychoactive substance abuse can pose to them, their passengers and other road users. Additionally, enforcement of laws on substance abuse control is recommended, as knowledge alone has been reported to be ineffective in overall substance abuse control.

The current study found a significant association between ethnicity, religion, monthly income, knowledge, perception, and psychoactive substance abuse. In consonance with our findings, ethnicity of commercial drivers have reportedly exerted significant influence on their usage of psychoactive substances as established by other studies [32, 34, 35]. This may be a pointer to the need to clarify the relationship between socio-cultural elements and the abuse of psychoactive substances among drivers. Previous studies conducted in Nigeria and Ethiopia have also shown religion, age, and marital status as socio-demographic variables influencing the abuse of psychoactive substances among commercial drivers [4, 20, 32]. Drivers who were Islamic religious followers were five times more likely to use psychoactive substances than those practicing Christianity. This finding agreed with a study conducted in Modjo, Ethiopia, which found that drivers who were Christians were 48% less likely to use psychoactive substances than Muslim religious followers [4]. Religion and religious involvement are documented protective factors against the consumption and risky pattern of psychoactive substances use [36, 37]. This could suggest that the drivers’ religious affiliations may significantly influence their use of psychoactive substances. Similar to the result in this study, drivers with low monthly income are usually in need to earn more, so they have higher likelihood to hurriedly engage in frequent trips and may be prone to psychoactive substance abuse with possibilities of higher road traffic crashes as also documented by other studies [4, 35]. Discordant findings were however reported by Girotto et al. in Brazil whose study found a direct relationship between higher income and psychoactive substance abuse among drivers. The differences observed may be due to variations in the study setting and study participants used [38]. The poor knowledge and perception about the abuse of psychoactive substances among the study participants as observed in this study could be counter-productive in the fight against this menace. Empirical evidences have also shown relationships between poor knowledge and perception and the abuse of psychoactive substances among drivers [16, 39, 40]. Thus, concerted efforts to improve the knowledge and perception about usage of psychoactive substances and their deleterious effects among commercial drivers should be geared up.

Socio-demographic variables of drivers such as ethnicity, education and religion were not usually evaluated, as previous studies focused mainly on the relationship between substance abuse and road traffic accidents [32, 40, 41]. Socio-demographic characteristics, are therefore an integral aspect of the determinants of psychoactive substance abuse and an area of interest to explore in further research. Targeting commercial bus drivers for educational interventions could also help correct their poor perception about the abuse of these psychoactive substances and forestall future road traffic accidents.

### Study Limitations

The responses of the commercial bus drivers in this study were self-reported and have to be accepted as they were stated, the research assistants were well trained to minimize reporting bias. In addition to this, social desirability bias was envisaged and this was reduced by avoiding leading questions and using interval questions. We might not be able to generalize our findings to the whole country. However, the findings of our study could be extrapolated to the entire Southern part of Nigeria because of the similarities in socio-economic and cultural characteristics.

## Conclusion

Despite so many interventions instituted by the Federal Government of Nigeria (FGN) and Non-Governmental Organizations (NGOs), it is regrettable that the prevalence of psychoactive substances is still high among commercial bus drivers and, as such, constitutes a significant public health threat. Surprisingly, religion, knowledge, and perception about psychoactive substance remained the only statistically significant explanatory variables for the abuse of substances among the study participants. Through its agencies, such as the Federal Road Safety Corp, the government should target commercial bus drivers for both preventive and routine educational interventions through collaboration with the National Union of Road Transport Workers. In the same vein, religious leaders should be engaged in proper education to empower their congregants with relevant information about the use of psychoactive substances, knowing fully well that drivers are also part of the religious congregations. Access to alcohol and other psychoactive substances by commercial bus drivers in and around motor parks should be restricted by enactment of laws and enforcement by the National Drug Law Enforcement Agency (NDLEA) to drastically reduce RTCs drastically.

## Data Availability

Upon request, we can offer onsite access to external researchers to the data analyzed at the Directorate of Primary Health Care and Disease Control, Oyo State Primary Health Care Board, State Secretariat, Agodi, Ibadan. To do so, Dr. Olaniyan Akintunde Babatunde should be contacted.

## Abbreviations

RTCs: Road Traffic Crashes
RTAs: Road Traffic Accidents
FRSC: Federal Road Safety Corps
DUI: Driving Under the Influence of Drugs
UNODC: United Nations office on Drugs and Crime
NURTW: National Union of Road Transport Workers
LGAs: Local Government Areas

## Declarations

### **Competin interests**

The authors declared no competing interests.
